# miR-219-5p Inhibits Receptor Tyrosine Kinase Pathway by Targeting EGFR in Glioblastoma

**DOI:** 10.1371/journal.pone.0063164

**Published:** 2013-05-17

**Authors:** Soumya Alige Mahabala Rao, Arivazhagan Arimappamagan, Paritosh Pandey, Vani Santosh, Alangar Sathyaranjandas Hegde, Bangalore Ashwathnarayanara Chandramouli, Kumaravel Somasundaram

**Affiliations:** 1 Microbiology and Cell Biology, Indian Institute of Science, Bangalore, Karnataka, India; 2 Department of Neurosurgery, National Institute of Mental Health and Neuro Sciences, Bangalore, Karnataka, India; 3 Department of Neuropathology, National Institute of Mental Health and Neuro Sciences, Bangalore, Karnataka, India; 4 Sri SatyaSai Institute of Higher Medical Sciences, Bangalore, Karnataka, India; University of Central Florida, United States of America

## Abstract

Glioblastoma is one of the common types of primary brain tumors with a median survival of 12–15 months. The receptor tyrosine kinase (RTK) pathway is known to be deregulated in 88% of the patients with glioblastoma. 45% of GBM patients show amplifications and activating mutations in EGFR gene leading to the upregulation of the pathway. In the present study, we demonstrate that a brain specific miRNA, miR-219-5p, repressed EGFR by directly binding to its 3′-UTR. The expression of miR-219-5p was downregulated in glioblastoma and the overexpression of miR-219-5p in glioma cell lines inhibited the proliferation, anchorage independent growth and migration. In addition, miR-219-5p inhibited MAPK and PI3K pathways in glioma cell lines in concordance with its ability to target EGFR. The inhibitory effect of miR-219-5p on MAPK and PI3K pathways and glioma cell migration could be rescued by the overexpression of wild type EGFR and vIII mutant of EGFR (both lacking 3′-UTR and thus being insensitive to miR-219-5p) suggesting that the inhibitory effects of miR-219-5p were indeed because of its ability to target EGFR. We also found significant negative correlation between miR-219-5p levels and total as well as phosphorylated forms of EGFR in glioblastoma patient samples. This indicated that the downregulation of miR-219-5p in glioblastoma patients contribute to the increased activity of the RTK pathway by the upregulation of EGFR. Thus, we have identified and characterized miR-219-5p as the RTK regulating novel tumor suppressor miRNA in glioblastoma.

## Introduction

microRNAs (miRNAs) have emerged as one of important regulators of the interaction network that controls various cellular processes. miRNAs are short non-coding RNAs which regulate the target mRNA by binding mostly in the 3′-UTR and bring about either translational repression or degradation of the target. miRNAs are shown to play key roles in cell survival, proliferation, apoptosis, migration, invasion and various other characteristic features that get altered in human cancers [1]. miRNAs are characterized to have oncogenic or tumor suppressor role and the aberrant expression of miRNAs is reported in multiple human cancer types [2].

Astrocytomas are one of the most common types of primary brain tumors in humans [3]. Glioblastoma is the most aggressive of all grades of astrocytomas and has a very poor prognosis. The glioblastoma occurs either *de novo* (primary glioblastoma) or progression from the lower grades (secondary glioblastoma) [4]. As per the standard treatment protocols, the patients diagnosed with glioblastoma receive radiotherapy and concomitant temozolamide chemotherapy [5]. The median survival of glioblastoma patients is 12–15 months [6]. In order for the better understanding of the biology of glioblastoma, The Cancer Genome Atlas (TCGA) group has attempted to integrate large-scale multiplatform data and identified three core pathways (p53, pRb and RTK pathways) that were deregulated in glioblastoma [7]. In addition, based on the TCGA profiling studies, the glioblastoma samples were further divided into subtypes based on the marker profiles [8] and G-CIMP feature [9]. In spite of the new advancements in the field of glioblastoma research, the clinical outcome of the patients has not substantially changed over a period of time necessitating the in-depth elucidation of the regulation of pathways for the better understanding of the disease and also to develop personalized, more targeted therapies.

The increased aberrant activity of the receptor tyrosine kinase (RTK) pathway in glioblastoma is attributed to the deregulation of EGFR in 45% of glioblastoma patients [7]. The expression and activation of the receptors are reported to be deregulated by events like amplification and activating mutations. The aberrant expression of EGFR could also be due to the deregulation of miRNAs, which in the untransformed astrocytes regulate and fine-tune the levels of the RTKs. The regulation of EGFR by miRNAs is reported in several cancers including glioblastoma [10], [11].

In the current study, we have identified that miR-219-5p, one of the downregulated miRNAs in glioblastoma, targeted EGFR by binding to its 3′-UTR. We demonstrated that miR-219-5p acts as tumor suppressor by inhibiting proliferation, anchorage independent growth and migration of glioma cells. The overexpression of miR-219-5p also inhibited the activity of PI3K and MAPK pathways. The overexpression of wild type and vIII mutant forms of EGFR lacking the 3′-UTR rescued cells from the inhibitory effect of miR-219-5p on MAPK and PI3K pathways and also on glioma cell migration. In addition, we showed that glioblastoma patients with lower levels of miR-219-5p had increased EGFR protein levels as compared to those having higher levels of miR-219-5p. Our study demonstrated that miR-219-5p targeted EGFR in glioma cells and the downregulation of miR-219-5p in the glioblastoma patient samples could be attributed to the increased levels of EGFR, thus increasing the activity of the RTK pathway and promoting tumor growth.

## Materials and Methods

### Cell lines and tumor samples

The cell lines U138, U343, U251, U87, 293T and SW480 cells were kind gift from Dr. Abhijit Guha [12] and were grown in Dulbecco's modified Eagle medium (DMEM) supplemented with 10% FBS. U87 parental, U87 - wtEGFR stable cells and U87-ΔEGFR stable cells were kind gift from Dr. Frank Furnari, Ludwig Institute for Cancer Research, UCSD and were generated as described before [13]. The anaplastic astrocytoma and glioblastoma tumor samples were collected from patients who were operated at National Institute of Mental Health and Neurosciences (NIMHANS) and Sri Sathya Sai Institute of Higher Medical Sciences (SSSIHMS), Bangalore, India. As normal control brain sample, a portion of the non-dominant anterior temporal cortex resected during surgery for intractable epilepsy was used. The freshly received tissues from the neurosurgical operating rooms were bisected and one half was placed in RNA later (Ambion Inc., USA), stored at −70°C and used for RNA isolation. The other half was fixed in 10% buffered neutral formalin, processed for paraffin sections and was used for histopathology and immunohistochemistry (IHC) as described before [14]. The study has been scrutinized and approved by the ethics committee of the two clinical centers, NIMHANS and SSSIHMS, and written patient consent was obtained before initiation of the study as per the Institute Ethical Committee guidelines and approval. A total of 63 samples of malignant astrocytoma and 10 control brain tissues were used in the study. For microarray hybridization, a set of 46 samples comprising 13 anaplastic astrocytoma, 26 glioblastoma and 7 normal brain controls were used. For RT-PCR validation of miR-219-5p, we used an independent set of 24 glioblastoma and 3 normal brain controls.

### RNA isolation and miRNA Microarray

Total RNA isolation from the frozen tissue or cell lines and quantification were performed as described before [14]. The miRNA microarray profiling was done as described before [15].

### TaqMan Quantitative Real-Time PCR of the miRNAs

We used TaqMan real-time PCR method (Applied Biosystems) to measure the level of mature miR-219-5p. miRNA-specific cDNA was made using a specific stem–loop primer and a diluted reverse transcription product from 10 ng total RNA was used for real-time PCR reaction. Real-time PCR was performed using the ABI 7900HT real-time PCR system following the manufacturer's instructions. The small RNAs: U6, U44 and U48 were used as endogenous control. Delta Ct values were used for calculating the log 2 ratio. Statistical significance was tested by Mann–Whitney test using the GraphPad PRISM software.

### Transfection of miRNA Precursors

For the overexpression of miR-219-5p, the precursors, pre-miRs for miR-219-5p and negative control Pre-miRs were obtained from Ambion. Transfection was performed by adding the precursors at a concentration of 30 nM using the siPORT NeoFX transfection agent (Ambion) following the manufacturer's instructions. After transfection, RNA was isolated at 72 hrs and the overexpression of miRNA was confirmed by assaying for the level of mature miR-219-5p by TaqMan quantitative real-time PCR.

### Proliferation assay

U138, U343 and U251 cells were transfected with negative control pre-miR or pre-miRs for miR-219-5p and 10000 cells were plated in 24-well plates At different time intervals, the cells were trypsinized, suspended in PBS and viable cells were counted using hemocytometer. The statistical significance was calculated by Students' T test in MS excel.

### Colony formation assay

U343 cells were transfected with negative control pre-miR or pre-miR for miR-219-5p. After 36 hrs of transfection, cells were counted and 300 cells/well were plated in 12-well plates. The culture media was changed every 3 days. After 2-weeks of plating, the colonies were fixed using 70% ethanol and stained with 0.05% crystal violet and photographed. The statistical significance was calculated by Students' T test in MS excel.

### Anchorage independent growth by colony formation in soft agar

U138 and U251 cells were transfected with negative control pre-miR or pre-miRs for miR-219-5p. After 36 hrs of transfection, the cells were harvested and plated for soft agar assay. Base agar was prepared by using 800 µl of 0.6% agar in complete medium in a 6-well plate. After the base agar was set, agar solution was mixed with 5×10^3^ U138/U251 cells (to a final concentration 0.34%) and overlaid on the solidified base agar. The plates were incubated at 37°C for up to 3 weeks. Triplicate wells were prepared for each transfection. Colonies were counted and microphotographed. The statistical significance was calculated by Students' T test in MS excel.

### Migration assay

U138, U87 parental, U87 wt-EGFR stable and U87 vIII EGFR stable cells were transfected with negative control pre-miR or pre-miRs for miR-219-5p. After 72 hrs of transfection, migration assay was set up using the Control Inserts (24-well, Catalog # 354578) from BD Biosciences following the manufacturer's instructions. After 18 hrs of incubation, migrated cells were fixed, stained, photographed and counted as described before [16].

### Western Blotting

Cell lysates were prepared using RIPA buffer (Radioimmunoprecipitation assay buffer) after 72 hrs of pre-miR transfection. The lysates were subjected to SDS-PAGE followed by western blotting as described before [17]. The antibodies that were used in the study were: EGFR- Imgenex #IMG-90417-2, phospho-ERK1/2 - Cell Signaling #9101, total ERK1/2 - Cell Signaling #9102, phospho-Akt - Cell Signaling #4051, total Akt - Cell Signaling #9272, phospho-4EBP1 - Cell Signaling #9471, Tubulin - Calbiochem #CP06 and Actin - Sigma #A3854). The band intensity was quantified by using Multi Gauge software from Fujifilm and normalized for the levels of internal control proteins (actin or tubulin) of the corresponding lysates.

### Real time qRT-PCR

Real time qRT-PCR for EGFR using specific primers was performed as described before [18].

### 3′-UTR reporter plasmid

The reporter plasmid pGL3-EGFR 3′-UTR was kind gift from Dr. Benjamin Purow, University of Virginia Neuro-Oncology Center [10]. 3′-UTR reporter plasmids were constructed as described before [10] via insertion of the EGFR 3′-UTR sequence (1.7 kb) into the XbaI restriction site 3′ to luciferase in the pGL3-promoter plasmid (Promega Corp.). Mutation of miR-219-5p binding sites in pGL3-EGFR 3′-UTR was performed by site directed mutagenesis using the QuikChange® XL Site-Directed Mutagenesis Kit (Catalog #200516) from Stratagene following manufacturer's instructions. The primers used for site directed mutagenesis were: 5′-GCCCACATTGGATTCATCAGCATTGCCGCGGCAAGCCCACAGC-3′ and 5′-GCTGTGG GCTTGCCGCGGCAATGCTGATGAATCCAATGTGGGC-3′

### Luciferase assay

For 3′-UTR reporter luciferase assay, the cells were transfected with pre-miRs. After 36 hrs of pre-miR transfection, 3′-UTR reporter plasmid (1 µg) along with pCMV-beta plasmid (0.5 µg) was transfected using lipofectamine following the manufacturer's instructions. After 48 hrs of plasmid transfection, cell extracts were made and luciferase activity was measured and normalized for beta-galactosidase activity as described before [19].

### miRNA data from TCGA dataset

miRNA expression data for glioblastoma samples were downloaded from The Cancer Genome Atlas (TCGA) data portal and normalized as described before [20].

## Results

### The expression of miR-219-5p is downregulated in glioblastoma

**Figure 1:**
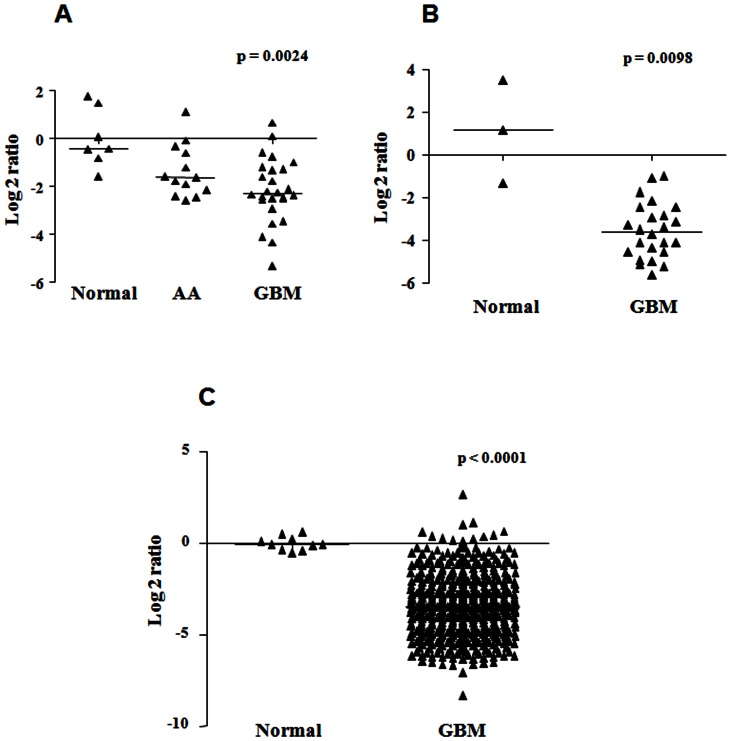
miR-219-5p is downregulated in glioblastoma. Expression of miR-219-5p in malignant astrocytoma [anaplastic astrocytoma (AA) and glioblastoma (GBM)] as compared to the control normal brain tissue (Normal) is plotted as scatter plot. **A.** From the miRNA microarray data, miR-219-5p expression was found to be significantly downregulated in anaplastic astrocytoma (AA; n = 13) and glioblastoma (GBM; n = 26) with a p value of 0.0024 (from Kruskal-Wallis test in One-Way ANOVA). **B.** In the real-time qPCR validation set, miR-219-5p expression was found to be significantly downregulated in glioblastoma (GBM; n = 24) with a p value of 0.0098 (from Mann-Whitney test). **C.** In the TCGA dataset, miR-219-5p expression was found to be significantly downregulated glioblastoma (GBM; n = 492) with a p value of <0.0001 (from Mann-Whitney test).

In order to understand the role of miRNAs in the development of glioblastoma, we were interested in the identification of the targets for the miRNAs that were aberrantly expressed in glioblastoma. From the previously performed miRNA expression profiling [15], we chose miR-219-5p, one of the miRNAs that showed highest downregulation in glioblastoma as compared to the control normal brain tissue [15]. miR-219-5p was shown to have brain specific expression [21]. In the miRNA microarray data, the expression of miR-219-5p was found to be significantly downregulated (p = 0.0024) in anaplastic astrocytoma (n = 13) by 3-fold (median log 2 ratio of −1.6) as well as in glioblastoma (n = 26) by 5-fold (median log 2 ratio of −2.3) when compared to the control normal brain tissue (n = 7) (**Figure 1A**). We further validated the downregulation of miR-219-5p by real-time qPCR in an independent cohort of glioblastoma patient samples. In the validation set, miR-219-5p expression was significantly downregulated (p = 0.0098) in glioblastoma patient samples (n = 24) by 12-fold (median log 2 ratio of −3.6) when compared to the control normal brain tissue (n = 3) (**Figure 1B**). We also analyzed the expression of miR-219-5p in the miRNA expression profiling data by The Cancer Genome Atlas (TCGA) group. In the TCGA dataset, miR-219-5p showed significant downregulation (p<0.0001) in glioblastoma patient samples (n = 492) by 10-fold (median log 2 ratio of −3.4) when compared to the normal control brain tissue (n = 10) (**Figure 1C**). The expression profiling of miR-219-5p thus confirmed that miR-219-5p expression levels were downregulated in glioblastoma patient samples indicating a potential role for miR-219-5p in the development of glioblastoma.

### Overexpression of miR-219-5p in glioma cell lines decreased proliferation, anchorage independent growth and migration of glioma cells

**Figure 2:**
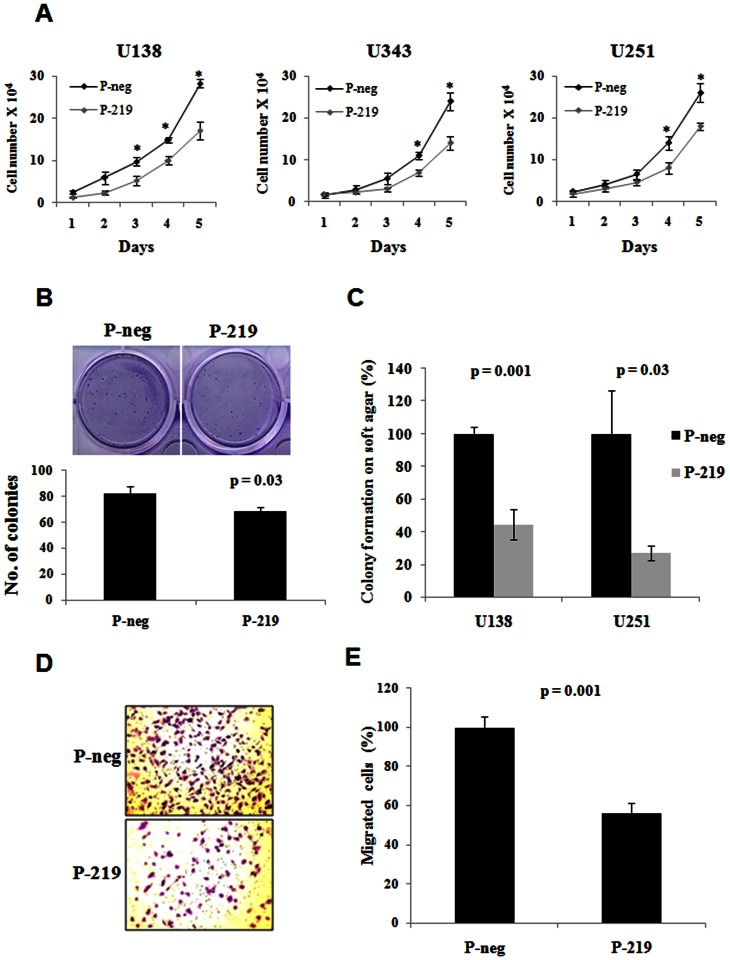
Overexpression of miR-219-5p in glioma cell lines decreased proliferation, anchorage independent growth and migration of glioma cells. **A.** U138, U343 and U251 cells were transfected with negative control pre-miRs (P-neg) or pre-miRs for miR-219-5p (P-219) and plated in triplicates in 24-well plates. The cell number was counted by using hemocytometer at indicated time points. Average cell number ± SD was plotted. p value was calculated by Students' t test in MS excel. * represents significant p value. For U138, the p values were 0.042, 0.006, 0.003 and 0.003 for Day 2, Day 3, Day 4 and Day 5 respectively; for U343, the p values were 0.06, 0.004, and 0.003 for Day 3, Day 4 and Day 5 respectively; for U251, the p values were 0.009, 0.01 for Day 4 and Day 5 respectively. **B.** U343 cells were transfected with negative control pre-miRs (P-neg) or pre-miRs for miR-219-5p (P-219) and 300 cells were plated in triplicates in 12-well plates. After 3 weeks of plating, the colonies were stained with crystal violet, photographed (top) and counted (bottom). Average colony number ± SD was plotted. p value was calculated by Students' t test in MS excel. **C.** U138 and U251 cells were transfected with negative control pre-miRs (P-neg) or pre-miRs for miR-219-5p (P-219). After 36 hrs of transfection, cells were re-plated on the soft agar plates in triplicates. After 3 weeks, the soft agar colonies were counted under microscope. Average colony number ± SD was plotted. p value was calculated by Students' t test in MS excel **D.** U138 cells were transfected with negative control pre-miRs (P-neg) or pre-miRs for miR-219-5p (P-219). After 72 hrs of transfection, cells were counted and plated for matrigel migration assay and after 18 hrs, cells were fixed, stained with crystal violet, photographed. **E.** Quantification of **D.** Average cell number ± SD was plotted. p value was calculated by Students' t test in MS excel.

Since miR-219-5p was downregulated in glioma, we assessed the effect of overexpression of miR-219-5p on proliferation, anchorage independent growth and migration of glioma cells. We overexpressed miR-219-5p in glioma cell lines using precursor mimics for miR-219-5p (P-219) along with negative control pre-miRs (P-neg). We confirmed the over expression of miR-219-5p by real time qPCR which showed that there was upregulation of miR-219-5p in cells transfected with pre-miRs for miR-219-5p (P-219) cells when compared the negative control pre-miR (P-neg) transfected cells (**Figure S1A**). The overexpression of miR-219-5p glioma cell lines resulted in significant reduction in the proliferation of U138, U343 and U251 cells (**Figure 2A**). In order to assess the long-term effect of miR-219-5p overexpression on glioma cell proliferation, we performed colony formation assay in U343 cells. The overexpression of miR-219-5p in U343 cells significantly decreased the number of colonies when compared to the negative control pre-miR transfected cells (**Figure 2B**). To assess the effect of miR-219-5p on anchorage independent growth of glioma cells, we overexpressed miR-219-5p and set up soft agar colony formation assay. When compared to the negative control pre-miR transfected cells, pre-miR-219-5p transfected cells showed significant reduction in the soft agar colony formation in U138 and U251 cell line (**Figure 2C**). The overexpression of miR-219-5p resulted in the significant decrease in the migration of U138 cells (**Figure 2D, E**). These results suggested that miR-219-5p plays growth inhibitory role in glioma development.

### miR-219-5p targets EGFR by directly binding to its 3′-UTR

**Figure 3:**
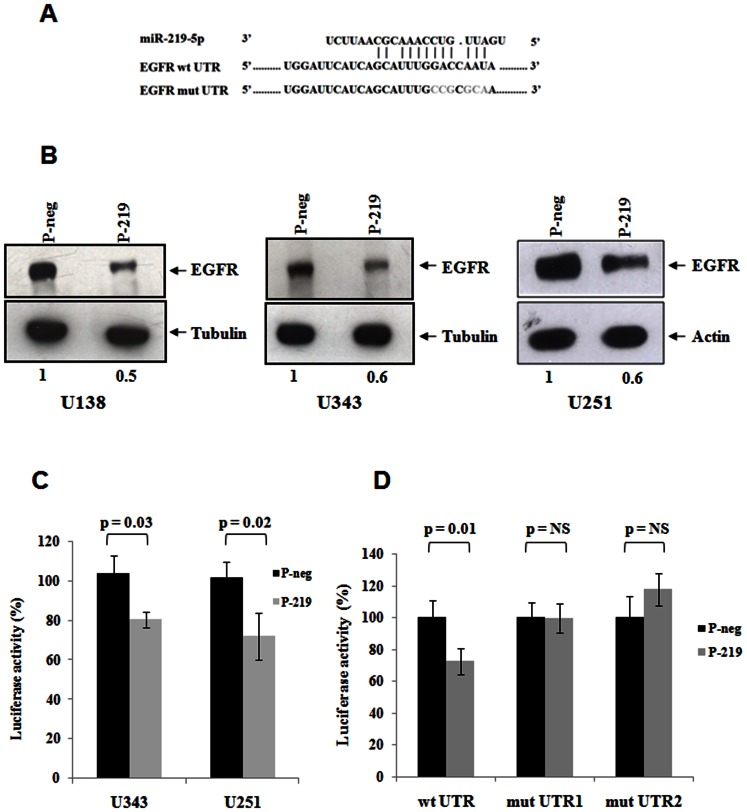
miR-219-5p targets EGFR. **A.** Predicted binding site for miR-219-5p in the 3′-UTR of EGFR. EGFR-mut-UTR shows the sequence of the mutated 3′-UTR. **B.** U138, U343 and U251 cell lines were transfected with Pre-miR-219 (P-219) or negative control pre-miRs (P-neg). After 72 hrs of transfection, western blot for EGFR was performed using the total cell lysate. **C.** U343 and U251 cell lines were transfected with pre-miRs for miR-219-5p (P-219) or negative control pre-miRs (P-neg). After 36 hrs of pre-miR transfection, pGL3-EGFR-UTR reporter plasmid was transfected and after 48 hrs, luciferase activity was measured. Mean luciferase activity ± SD was plotted. p value was calculated by Students' t test in MS excel. **D.** U251 cell line was transfected with Pre-miR-219 (P-219) or negative control pre-miRs (P-neg). After 36 hrs of pre-miR transfection either wild type pGL3-EGFR-UTR (wt UTR) or two clones of mutant pGL3-EGFR-UTR (mut1 and mut2) reporter plasmid was transfected and after 48 hrs, luciferase activity was measured. Mean activity ± SD was plotted and p value was calculated by Students' T test in MS excel. NS represents Non-Significant p value.

EGFR, one of the important upregulated RTKs in glioblastoma, was predicted as a target of miR-219-5p by the target prediction algorithm RNA Hybrid, which proposed binding site for miR-219-5p in the 3′-UTR of EGFR (**Figure 3A**). To experimentally validate the prediction, we overexpressed miR-219-5p in glioma cell lines using precursor mimics for miR-219-5p (P-219) along with negative control pre-miRs (P-neg). Upon overexpression of miR-219-5p in glioma cell lines, there was consistent 40–50% reduction in the protein levels of EGFR in U138, U343 and U251 cell lines as assayed by western blotting (**Figure 3B**). The transcript levels of EGFR did not change upon over expression of miR-219-5p (**Figure S1B**) indicating that miR-219-5p exerted translation repression of EGFR transcript without the degradation of the target transcript. Further to investigate whether miR-219-5p directly binds to the 3′-UTR of EGFR, we used a reporter plasmid having full length 3′-UTR of EGFR cloned downstream to the luciferase gene. Upon the overexpression of miR-219-5p, there was significant reduction in the luciferase activity in U343 and U251 cell lines (**Figure 3C**) showing the direct interaction of miR-219-5p with the 3- UTR of EGFR transcript. Further to check the specificity of the predicted binding site for miR-219-5p, we mutated the bases that were important for the binding of the seed region of the miRNA in the reporter plasmid. When the clones of mutated plasmid were transfected into U251 cell line, the overexpression of miR-219-5p did not have any inhibitory action on the luciferase activity as against that exerted on the wild-type plasmid (**Figure 3D**). The same effects were also seen in 293T and SW480 cell lines (**Figure S1C, D**). This indicated that the predicted binding sites were indeed important for the miR-219-5p - EGFR transcript interaction.

### Overexpression of miR-219-5p reduced the activation of PI3 Kinase and MAP kinase pathways

**Figure 4:**
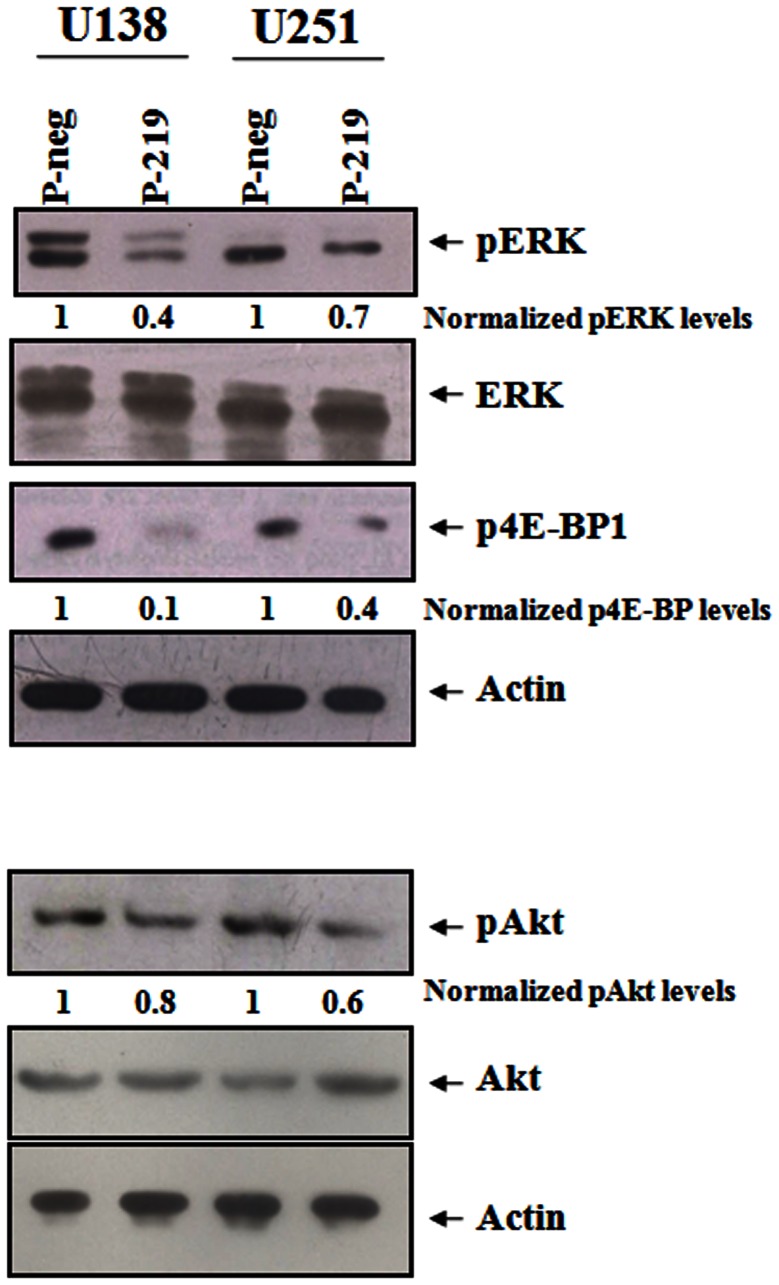
Overexpression of miR-219-5p reduced the activities of MAPK and PI3K pathways. U138 and U251 cells were transfected with pre-miR-219-5p (P-219) or negative control pre-miRs (P-neg). After 72 hrs of pre-miR transfection, cell lysate was prepared and subjected to the western blotting for phospho-ERK 1/2 (Tyr202/Tyr204) and total ERK 1/2; phospho-Akt (Ser473), total Akt and phospho-4E-BP1 (Ser65).

Upon ligand binding, the receptor tyrosine kinase EGFR activates two pathways: PI3K and MAPK pathways [4]. Since miR-219-5p targeted EGFR and was downregulated in glioblastoma, we looked at the effect of miR-219-5p overexpression on these pathways. In U138 and U251 cell lines, the overexpression of miR-219-5p resulted in the decreased activity of MAP kinase pathway as assayed by decreased phosphorylation of Tyr202/Tyr204 of ERK1/2 without any change in the levels of total ERK1/2 in both U138 (reduction by 60%) and U251 (reduction by 30%) cell lines (**Figure 4**). Similarly, overexpression of miR-219-5p resulted in the decreased activation levels of PI3 kinase pathway as assayed by the decreased phosphorylation of Ser473 of Akt (reduction by 20% and 40% in U138 and U251 cell lines respectively) without any change in the levels of total Akt (**Figure 4**) as well as the decreased phosphorylation of Ser65 of 4E-BP1 both U138 (reduction by 90%) and U251 (reduction by 60%) cell lines (**Figure 4**). These results suggested that the overexpression of miR-219-5p indeed inhibited the activities of PI3K and MAPK pathways and the down regulation of miR-219-5p in glioblastoma patient samples could be contributing to the increased activity these pathways thus promoting the tumor growth.

### Overexpression of EGFR lacking 3′-UTR rescues the inhibitory effect exerted by miR-219-5p on PI3K/MAPK pathways and cell migration

**Figure 5:**
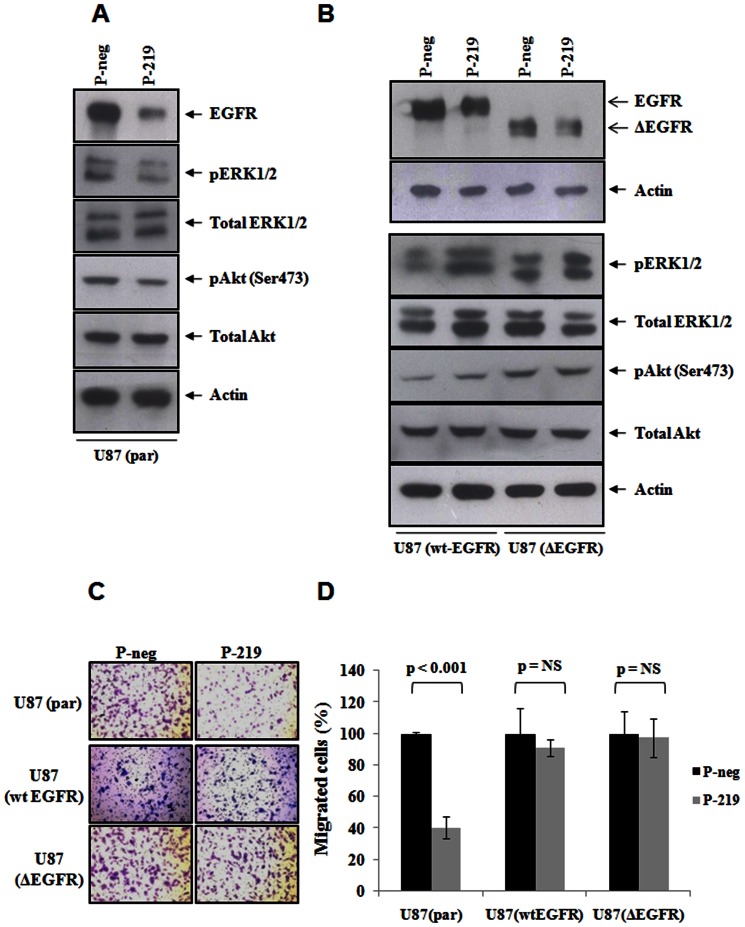
Inhibition of MAPK/PI3K pathways and glioma cell migration by miR-219-5p are mediated by EGFR. U87 parental (par) (**A**), U87 (wt-EGFR) and U87 (ΔEGFR) (**B**) cells were transfected with pre-miR-219-5p (P-219) or negative control pre-miRs (P-neg). After 72 hrs of pre-miR transfection, cell lysate was prepared and subjected to the western blotting for EGFR, phosphor-ERK1/2 (Tyr202/Tyr204) and total ERK1/2; phosphor-Akt (Ser473), total Akt. **C.** U87 parental (par), U87 wt-EGFR cells and U87 (ΔEGFR) were transfected with negative control pre-miRs (P-neg) or pre-miRs for miR-219-5p (P-219). After 72 hrs of transfection, cells were counted and plated for matrigel migration assay and after 18 hrs, cells were fixed, stained with crystal violet, photographed. **D.** Quantification of **C.** Average cell number ± SD was plotted. p value was calculated by Students' T test in MS excel. NS represents Non-Significant p value.

It was of our interest to see if the inhibition of PI3K/MAPK pathways and glioma cell migration achieved by the overexpression of miR-219-5p is because of its ability to target EGFR. To address this, we made use of U87 glioma parental cell line along with its two derivatives U87-wtEGFR (that stably overexpress 3′-UTR-less wild type EGFR) and U87-ΔEGFR (that stably overexpress 3′-UTR-less vIII variant of EGFR). The overexpression of miR-219-5p resulted in the efficient reduction of EGFR (by 40%) only in U87-parental cell line (**Figure 5A**), but not in U87-wtEGFR and U87-ΔEGFR cell lines (**Figure 5B**). As observed in U138 and U251 cell lines before, overexpression of miR-219-5p in U87 parental cells resulted in decreased activity of MAPK and PI3K pathways as assayed by the reduced levels of pERK and pAkt (**Figure 5A**). The overexpression of miR-219-5p did not decrease the pERK and pAkt levels in U87-wtEGFR and U87-ΔEGFR cell lines (**Figure 5B**) suggesting that inhibition of MAPK and PI3K pathways by miR-219-5p could be rescued by the overexpression of EGFR lacking 3′-UTR. This indicated that effect of miR-219-5p on MAPK and PI3K signaling was because of its ability to target EGFR. We also looked at the effect of miR-219-5p on cell migration of U87-parental and U87-wtEGFR and U87-ΔEGFR cell lines. While overexpression of miR-219-5p resulted in the reduced migration of U87-parental cells (60% decrease; p<0.001), it did not have any effect on migration of U87-wtEGFR and U87-ΔEGFR cell lines (**Figure 5C, 5D**) which indeed suggested that the inhibitory effect of miR-219-5p on glioma cell migration was because of its ability to target EGFR.

### Correlation between miR-219-5p and EGFR protein levels in glioblastoma patient samples

**Figure 6:**
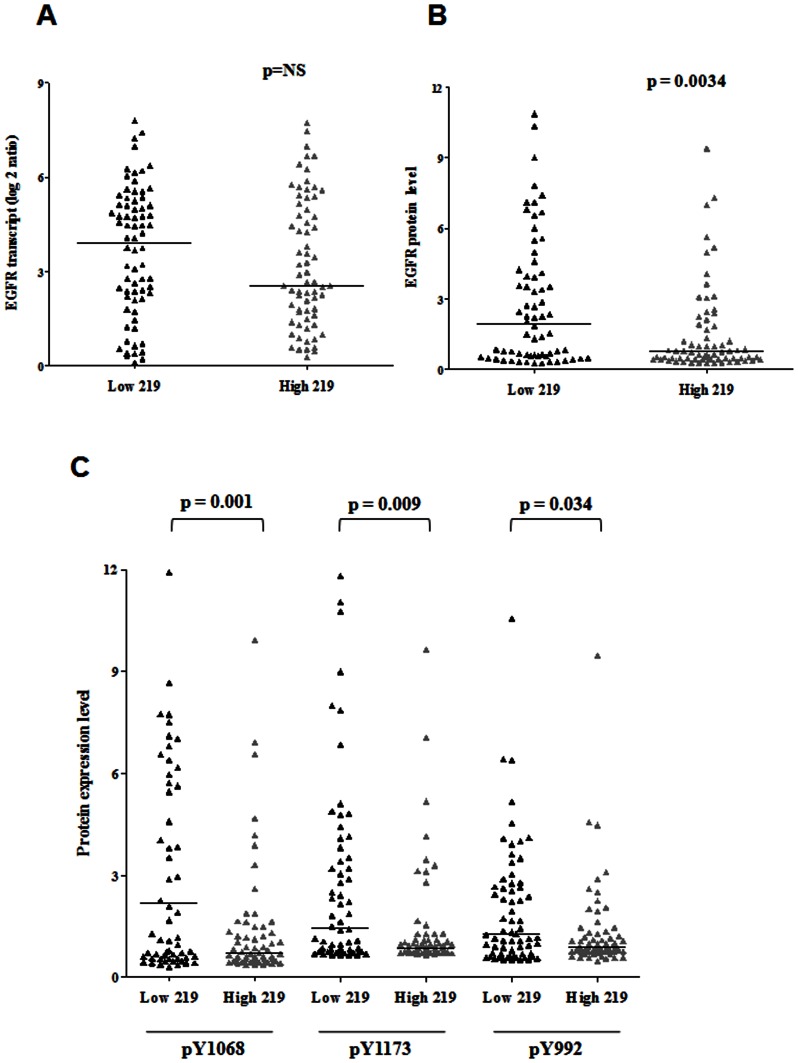
EGFR protein levels negatively correlated with miR-219-5p expression levels in glioblastoma samples. TCGA glioblastoma samples were divided into Low 219 (n = 66) and High 219 (n = 69) groups keeping median value for miR-219-5p as cut off. The EGFR transcript (**A**), total EGFR protein levels (**B**) and the protein levels of phosphorylated EGFR at Tyr1068, Tyr1173 and Tyr992 (**C**) were plotted as scatter plot for each group. p value was calculated by Mann-Whitney test in GraphPad Prism software.

Since our *in vitro* studies demonstrated that miR-219-5p targeted EGFR, we analyzed the correlation between miR-219-5p levels and EGFR protein levels in glioblastoma patient samples. To compare miR-219-5p and EGFR protein levels, we made use of the proteomics data available from the TCGA dataset. We divided patient samples into two groups - Low 219 (n = 66) and High 219 (n = 69) - based on the level of miR-219-5p expression (keeping the median expression value as cut-off). While EGFR transcript levels did not show any significant difference (**Figure 6A**), the patients with lower levels of miR-219-5p (Low 219 group) were found to have significant 2.5-fold higher levels of EGFR protein level as compared to those patients with higher levels of miR-219-5p (High 219) (p = 0.0034) (**Figure 6B**). It was of our interest to see the phosphorylation levels of EGFR at three different residues: Tyr1068, Tyr1173 and Tyr992. These residues are located in the carboxy terminus of EGFR and are the major sites of autophosphorylation which occurs as a result of EGF binding [22]. The phosphorylations of these sites mediate the binding of adaptor proteins like GRB which are the downstream effectors of the signaling. Patient sample with low levels of miR-219-5p showed significant increase in the phosphorylation levels of Tyr1068 (3.01 – fold; p = 0.001), Tyr1173 (1.67 – fold; p = 0.0097) and Tyr1173 (1.42 – fold; p = 0.034) as compared to patients having higher levels of miR-219-5p (**Figure 6C**). Thus, not only total EGFR but also activated phospho-EGFR levels negatively correlated with the miR-219-5p expression levels. This indicated that miR-219-5p downregulation in glioblastoma leads to the increased levels of EGFR.

## Discussion

There has been an increasing accumulation of literature in recent years reporting the deregulation of miRNAs in glioma. The importance of the deregulation of the miRNAs is demonstrated by the identification of the target genes that they regulate. Studies also have suggested the importance of miRNA expression profiling in glioma in their utility as diagnostic and prognostic signatures [23]. The receptor tyrosine kinase pathway in glioblastoma is reported to be over activated by events like amplification and activating mutations of EGFR [7]. The aberrant expression of miRNAs could be an important event in the deregulation of the receptor tyrosine kinase pathway. miR-7 was reported to target EGFR and IRS2 by directly binding to their 3′-UTRs and thereby regulated the pAkt levels in glioblastoma [10]. Recently, miR-128 was also shown to target EGFR and PDGFRα in glioma stem cells [11].

The miRNA microarray profiling done previously in the lab identified that the expression of miR-219-5p was downregulated in malignant astrocytoma when compared to the control normal brain tissue. The miR-219-5p is an intergenic miRNA encoded by two loci: one on chromosome 6 and another locus on chromosome 9 and it showed brain specific expression [21]. The downregulation of miR-219-5p in meningioma patients was associated with advanced clinical stages of meningioma and correlated with higher recurrence rates [24]. In addition, in an attempt to uncover the regulatory networks of microRNAs and transcription factors, miR-219-5p was one of the six miRNAs that were predicted to play key role in glioblastoma by topological and functional analyses of the subnetworks [25].

The overexpression of miR-219-5p in glioma cell lines resulted in the significant reduction in the proliferation, anchorage independent growth and migration of glioma cells *in vitro* suggesting the growth inhibitory role of miR-219-5p in glioblastoma. The target prediction algorithms identified the binding sites for miR-219-5p in the 3′-UTR of EGFR. Following the prediction, we indeed experimentally confirmed that miR-219-5p could target EGFR by directly binding to the 3′-UTR and brought about the translational repression of EGFR transcript in glioma cell lines. We also confirmed the binding sites for miR-219-5p in the 3′-UTR of EGFR by site directed mutagenesis of the important sites involved in binding, the mutation of which abolished the inhibitory activity achieved by the overexpression miR-219-5p. Previously, miR-219 was found to regulate the levels of Sox6, Hes5, PDGFRα, FoxJ3 and ZFP238 during oligodendrocyte differentiation [26], [27]. In T-cell lymphoma carrying the NPM-ALK fusion protein (ALK-TCL), miR-219 was found to target cell-stimulatory receptor ICOS (inducible T-cell co-stimulator or CD278) which was known to promote the proliferation of T-cells [28]. In hepatocellular carcinoma, miR-219-5p was found to be downregulated and was found to target glypican-3 (GPC3) which play role in cell division [29].

The overexpression of miR-219-5p resulted in the decreased activity of both PI3K and MAPK pathways suggesting that the down regulation of miR-219-5p could be contributing to the increased activity of these pathways in glioblastoma. The overexpression of EGFR could rescue the inhibitory effect of miR-219-5p on MAPK/PI3K pathways and also on glioma cell migration. In glioblastoma patient cohort, samples with lower levels of miR-219-5p showed increased levels of total as well as activated phosphorylated form of EGFR.

Our finding that the glioblastoma downregulated miR-219-5p targets EGFR provides insights into the additional mechanisms of deregulation of receptor tyrosine kinases in glioblastoma leading to the aberrant activation of the RTK pathway. The modulation of miR-219-5p in *in vivo* studies in glioma models might throw light on the utility of restoration of tumor suppressor miR-219-5p as therapeutic modality for glioblastoma.

## Supporting Information

Figure S1 **miR-219-5p targets EGFR by directly binding to its 3′ UTR.**
(PPTX)
