# Supplementary material for: miR-219-5p Inhibits Receptor Tyrosine Kinase Pathway by Targeting EGFR in Glioblastoma
Source: PLoS One. 2013 May 17;8(5):e63164. doi: 10.1371/journal.pone.0063164 (PMC3656853; doi:10.1371/journal.pone.0063164)
Supplement: Figure S1 — miR-219-5p targets EGFR by directly binding to its 3′ UTR. (PPTX) [file pone.0063164.s001.pptx]

## Slide 1
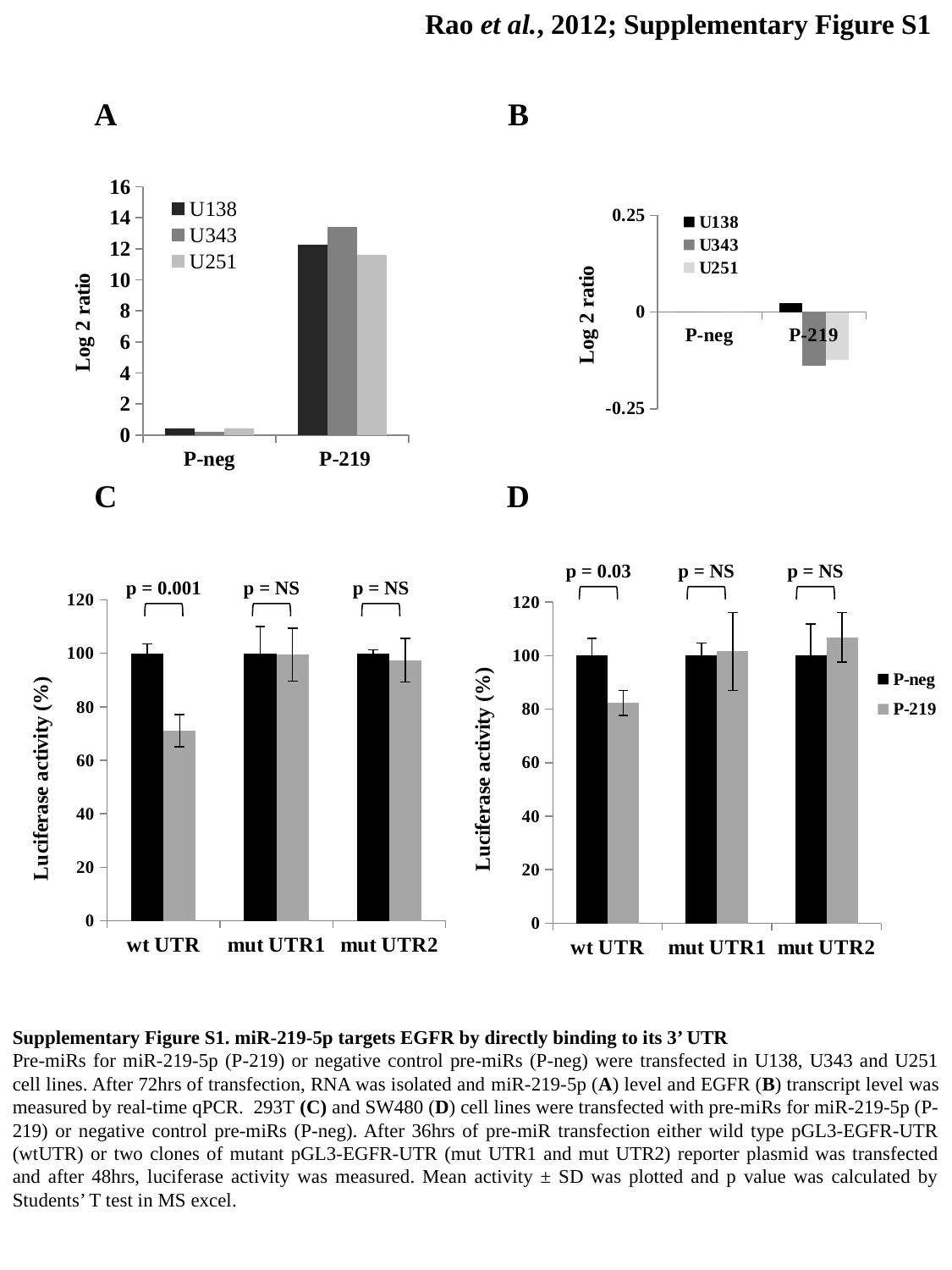

Rao et al., 2012; Supplementary Figure S1
A
B
### Chart
| Category | | | |
|---|---|---|---|
| P-neg | 0.42030458333333925 | 0.22300000000000061 | 0.4110000000000003 |
| P-219 | 12.288900166666663 | 13.43 | 11.6 |
### Chart
| Category | U138 | U343 | U251 |
|---|---|---|---|
| P-neg | 0.0 | 0.0 | 0.0 |
| P-219 | 0.02351074999999993 | -0.1395427500000024 | -0.12273487500000323 |C
D
p = 0.03
p = NS
p = NS
p = 0.001
p = NS
p = NS
### Chart
| Category | | |
|---|---|---|
| wt UTR | 100.0 | 71.10385471921772 |
| mut UTR1 | 100.0 | 99.45151650668072 |
| mut UTR2 | 100.0 | 97.43120274598252 |
### Chart
| Category | | |
|---|---|---|
| wt UTR | 100.0 | 82.3035209550901 |
| mut UTR1 | 100.0 | 101.5577143857706 |
| mut UTR2 | 100.0 | 106.8056762622204 |Supplementary Figure S1. miR-219-5p targets EGFR by directly binding to its 3’ UTR
Pre-miRs for miR-219-5p (P-219) or negative control pre-miRs (P-neg) were transfected in U138, U343 and U251 cell lines. After 72hrs of transfection, RNA was isolated and miR-219-5p (A) level and EGFR (B) transcript level was measured by real-time qPCR. 293T (C) and SW480 (D) cell lines were transfected with pre-miRs for miR-219-5p (P-219) or negative control pre-miRs (P-neg). After 36hrs of pre-miR transfection either wild type pGL3-EGFR-UTR (wtUTR) or two clones of mutant pGL3-EGFR-UTR (mut UTR1 and mut UTR2) reporter plasmid was transfected and after 48hrs, luciferase activity was measured. Mean activity ± SD was plotted and p value was calculated by Students’ T test in MS excel.
